# Misrepresentation of semaglutide in social media

**DOI:** 10.1007/s00210-025-04403-5

**Published:** 2025-07-19

**Authors:** Lara Elisabeth Propfe, Roland Seifert

**Affiliations:** Institute of Pharmacology, Hannover Medical School, Carl-Neuberg-Str. 1, D-30655 Hannover, Germany

**Keywords:** Semaglutide, Social media, Ozempic, Wegovy, GLP-1 receptor agonist, Weight loss, Type 2 diabetes mellitus

## Abstract

In recent years, semaglutide has repeatedly attracted attention in the press and on social media due to its effects in weight management. The social media hype led to off-label use and global supply shortages. Our goal was to identify differences in the presentation of semaglutide on individual social media platforms. Additionally, we aim to investigate the mentioning of various aspects such as adverse drug reactions, drug indications, supply shortages, and costs. Current social media trends regarding semaglutide were also included in the analysis. We collected semaglutide-related posts from the social media platforms Instagram, TikTok, X, Facebook, and YouTube. The posts were then analysed and compared with the adverse drug reactions presented in the STEP trials. Semaglutide was presented by social media users almost exclusively for weight reduction. Misrepresentation of the pharmacological properties of semaglutide with respect to mechanism of action, indication, adverse effects, and drug interactions was very common on social media. The important aspect of treatment costs and supply shortages was barely covered, if at all. Most content creators on Instagram and TikTok were female semaglutide users without medical knowledge. Strikingly, the very common gastrointestinal problems during semaglutide therapy were not even mentioned on Instagram. Some of the posts found under the hashtag “ozempic” advertised products that did not contain semaglutide. Violations of the Medicinal Products Act were also identified. Videos of weight loss results have a high probability of artificial intelligence (AI) modification, which can lead to user deception. Social media often provides misleading information, especially regarding drug indications and adverse drug reactions. YouTube has emerged as the most reputable platform for medical information, whereas Instagram provides the least medical knowledge. Misinformation and AI-modified videos can negatively impact the self-perception of young social media users, leading to unreflected medication use.

**Supplementary information:**

The online version contains supplementary material available at 10.1007/s00210-025-04403-5.

## Introduction

The GLP-1 receptor agonist semaglutide was initially approved by the FDA in 2017 (Stewart 2025) and was subsequently approved by the EMA in 2018 (Novo Nordisk 2018). It can be administered once a week as a subcutaneous injection or taken daily as a tablet. Semaglutide has initially been approved under the brand name “Ozempic” for the treatment of type 2 diabetes mellitus. In 2020, oral semaglutide was approved under the brand name “Rybelsus” for the treatment of type 2 diabetes. The oral daily dose of 14 mg is equivalent to 0.5 mg of subcutaneous semaglutide every week.

Studies have demonstrated that individuals experienced a mean weight reduction of approximately 12% over the course of treatment (Tan, 2022). As a result, it was also approved for weight reduction in 2022 under the trade name “Wegovy” (Rote Liste 2024a, b, c, d). In Germany, the GKV (social health insurance) does not cover the costs for semaglutide for people without type 2 diabetes mellitus because it is classified as a lifestyle drug that primarily improves quality of life (Martin 2024). Consequently, people seek to obtain Ozempic and Wegovy through fake prescriptions or via Internet advertisements. The use of semaglutide is contraindicated in people with type 1 diabetes mellitus, renal insufficiency, acute pancreatitis, and diabetic gastroparesis.

**Fig. 1:**
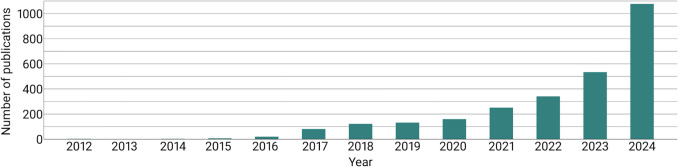
Chronological development of publications for the search term “ozempic” on PubMed.gov (retrieved on March 29, 2025)

In recent years, there has been an increasing number of posts on social media about semaglutide, especially about Ozempic. For example, on Instagram, there are 306,000 posts with the hashtag “ozempic” and 301,000 posts with the hashtag “semaglutide” (March 2025). Semaglutide has gained a lot of popularity not only on Instagram but also on other social media platforms like TikTok and YouTube. Some of the users share their experience with or results of taking semaglutide, while others simply share their personal opinion on the topic. There are also many healthcare professionals who explain the mechanisms of the drug and tell the subscribers about adverse drug reactions. The significance of the topic is also reflected in literature. As demonstrated in Fig. 1, there has been a notable increase in publications related to “ozempic” on PubMed.

Semaglutide has various positive effects on the human organism. It regulates the blood glucose concentration, controls the appetite and body weight, reduces major cardiovascular events, and reduces kidney-related events (Pantanetti et al. 2024; Perkovic et al. 2024). Despite the multitude of positive effects, there are also serious adverse drug reactions that can occur during the treatment. The most prevalent adverse events associated with semaglutide treatment include gastrointestinal disorders, hypoglycaemia, and diabetic retinopathy. Additionally, it is still unclear if semaglutide treatment can cause pancreatitis, pancreatic cancer, and thyroid cancer (Smits and Van Raalte, 2021). Further research must be done on the long-term effects of the medication.

A significant proportion of the global population is affected by type 2 diabetes mellitus. The global prevalence of this condition is estimated to be over 420 million, with a significant proportion of cases remaining undiagnosed or unreported. Type 2 diabetes mellitus is the most common form of diabetes, with 90% of the people with diabetes suffering from type 2 diabetes mellitus. The prevalence of diabetes in Germany is 7.4%, and in the USA, it is 8.8% (DBL-diabetes 2022). Notably, type 2 diabetes mellitus is becoming increasingly common among adolescents and young adults. It is assumed that type 2 diabetes in young adults leads to more complications and a reduced quality of life compared to late-onset diabetes (Lascar et al. 2018). The most significant risk factor for the development of type 2 diabetes is obesity.

The eligibility for semaglutide treatment in individuals with obesity is determined based on BMI. Wegovy is approved for people with a BMI of 27 kg/m^2^ and above, in conjunction with at least one weight-related comorbidity. Additionally, it is indicated for people with a BMI of 30 kg/m^2^ and above, without any comorbidities. In 2017, the average BMI of the US population was 27.7 kg/m^2^, and it is predicted to increase over time. In 2023, 40.3% of the US population was obese (Radtke 2025). Obesity is a global health problem that is associated with many secondary diseases such as coronary heart disease, major depressive disorder, and sleep apnoea (Apovian 2016). The prevention and treatment of obesity and type 2 diabetes mellitus therefore assume a major role in pharmacotherapy.

Two recent studies analysed the representation of semaglutide on a specific social media platform (Wantuch and Singleton, 2025; Campos-Rivera et al. 2025). This study is the first to examine differences in the representation of semaglutide between the individual social media platforms. The information contained in the analysed posts is then compared with scientific findings on semaglutide. These include the areas of application, adverse drug reactions, and general information about the drug.

Statistical parameters of the analysed social media platforms are shown in Table 1. All of the information displayed in the table were taken from data provided by the Statista Research Department in 2025. Monthly active users are defined as individuals who utilise the platform at least once within a 30-day period. With 3.07 billion monthly active users, Facebook is the world’s most popular social media platform. It was founded in 2004 and is owned by the company Meta Platforms. YouTube is the second most popular social media platform, with 2.53 billion monthly active users, and was founded in 2005. Since 2020, YouTube has offered users a new feature called YouTube Shorts, which allows them to create short-form videos similar to those on Instagram or TikTok. Other popular social media platforms include Instagram and WhatsApp, with two billion users each, and TikTok with 1.59 billion users. X (formerly Twitter) has a monthly active user base of 650 million users (Statista Daily Data, Statista Research Department 2025).

**Table 1:** Social media demographics in 2024 (Statista Daily Data, Statista Research Department 2025)

	**Female (%)**	**Male (%)**	**Monthly active users**	**Main age group**
Instagram	47	53	2 billion	< 25 years
TikTok	44	56	1.59 billion	< 25 years
YouTube	46	54	2.53 billion	25–34 years
Facebook	43	57	3.07 billion	25–34 years
X	36	64	650 million	25–34 years

Eighty percent of German Internet users claim to be active on social media platforms, and on average, people spend 18.7 h per week on social networks (Krah 2024). YouTube is used equally by users of all ages. In contrast, Instagram, TikTok, Facebook, and X are mainly used by adolescents and young adults. On Instagram and TikTok, almost 70% of the users are below the age of 35. The gender distribution on the platforms is relatively balanced, with male users slightly outnumbering female users. The difference is greatest on X, where the proportion of female users is 36%, while male users account for 64% (Statista Daily Data, Statista Research Department 2025).

Our present study aims to identify differences in the presentation of semaglutide on the most popular social media platforms to gain a better understanding of their credibility with regard to pharmacological content. Additionally, we intend to examine current social media trends associated with semaglutide.

## Materials and methods

**Fig. 2:**
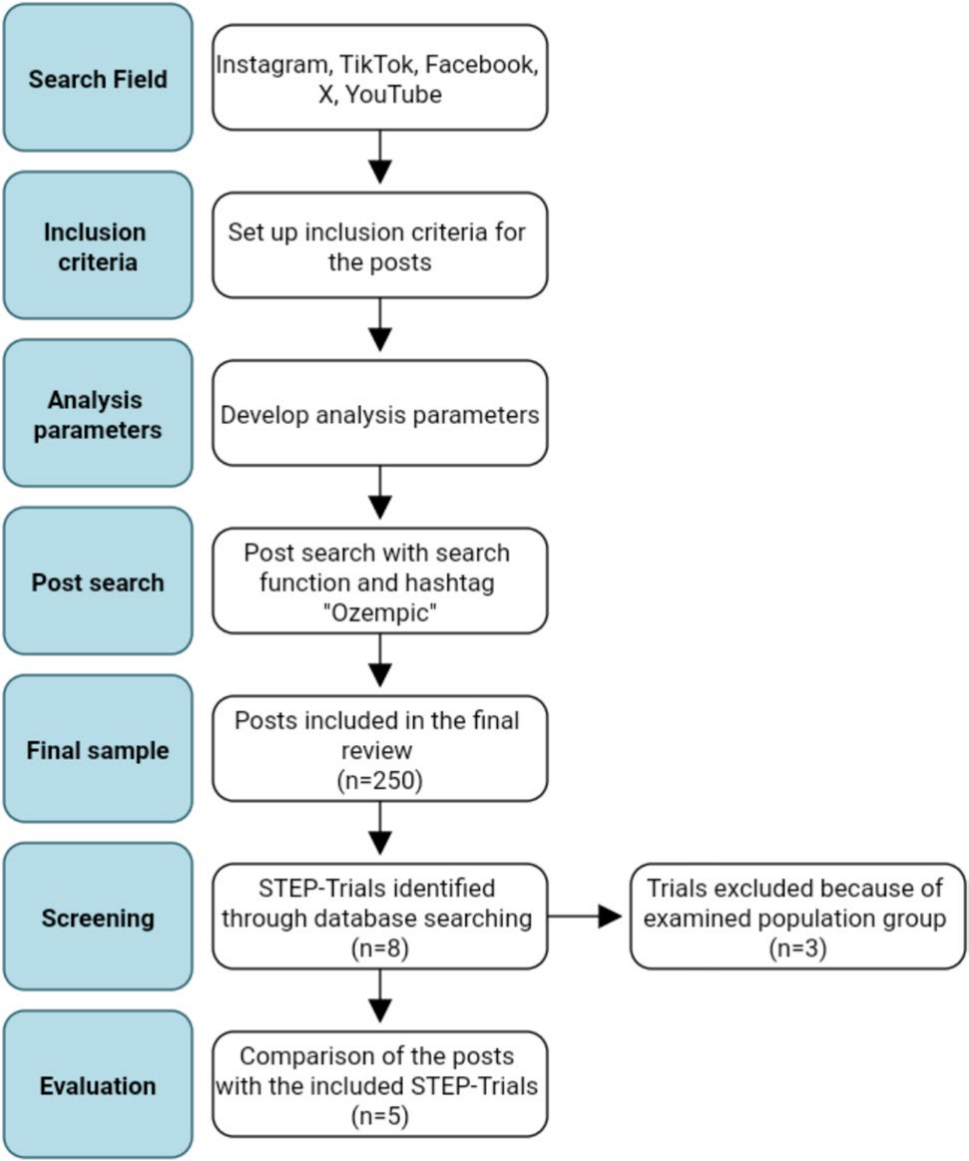
Flow chart of the process for quantitative sample extraction

All information about semaglutide on social media was extracted from the posts. These posts are publicly accessible. Figure 2 shows a flow chart of the process for quantitative sample extraction and comparison with the STEP trials. Due to their popularity and similar post formats, Instagram, TikTok, Facebook, X, and YouTube were selected for data collection. Subsequently, mandatory inclusion criteria were established. The following criteria were applied to narrow down the search: only public accounts were considered; the content was in German or English; and it had at least 1000 views. In instances where the number of views was not specified, only posts with at least 1000 likes were included in the study. Furthermore, analysis parameters were developed to enable the classification of the posts. The following analysis parameters were established: number of views, shares, likes and comments, type of post, creator, gender, language, objective of use, dosage form, information on adverse drug reactions, drug mechanism, indications, supply shortages, brand names, and costs.

The search function of the respective social media platform was used to identify relevant posts. The YouTube Shorts subcategory was used for the dissemination of content on the corresponding platform. The posts were found using the hashtag “ozempic”. To minimise potential data distortion resulting from previous user behaviour, a new account was created for the purpose of searching the platforms. The initial 50 articles displayed on each platform that satisfied the search criteria were incorporated into the analysis. Consequently, the final sample was comprised of 250 posts from five social media platforms. The data and links for each post were systematically documented in a table. Supplemental Table S1 summarises the basic characteristics of the posts. Some social media platforms (Instagram, TikTok, Facebook, YouTube) do not display the number of views or shares publicly. As a result, this data could not be collected.

The data was then subjected to analysis, using the aforementioned parameters, and subsequently evaluated. The adverse drug reactions stated in the posts were then compared with results from the STEP trials (Semaglutide Treatment Effect in People with Obesity). STEP-2, STEP-6, and STEP-TEENS were excluded because the study population did not align with the user base. The STEP-2 trial was excluded from the analysis due to its focus on individuals with obesity and type 2 diabetes, which differs from the demographic of the user base in social media. The STEP-6 trial investigated the effect of semaglutide in the East Asian population, whereas the analysed posts were predominantly sourced from the USA and Germany. The STEP-TEENS trial comprised adolescents aged 12 to 17 years as study participants. Given that Facebook is the only social media platform that accepts adolescent users aged 13 years and older, a strong deviation of the study population from the social media demographics is to be expected.

## Results

### Creators

**Fig. 3:**
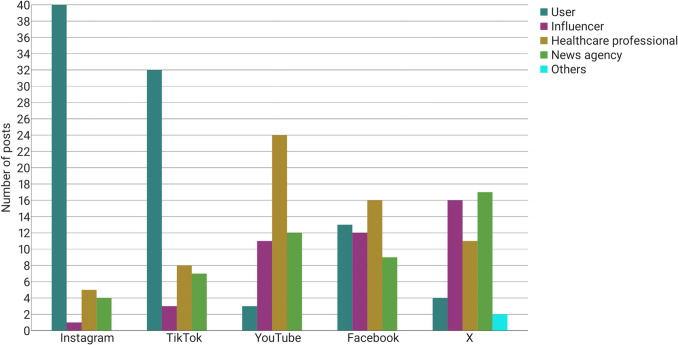
Creators of all posts as a bar chart

Figure 3 shows the creators of the posts. Eighty percent of the posts found on Instagram and 64% of the posts found on TikTok were created by users who take semaglutide. The content creators talk about their personal experiences with the medication or present images depicting their physical state both prior to and following the administration of Ozempic. The remaining 20% on Instagram and 36% on TikTok, respectively, were posted by healthcare professionals, news agencies, and influencers. In contrast, only 6% of the posts on YouTube and 8% of the posts on X were created by users who take Ozempic. A significant proportion of the content on YouTube (48%) was created by healthcare professionals. The remaining 46% of the posts on YouTube were posted by influencers (22%) and news agencies (24%).

The majority of posts on X were created by influencers or news agencies (66%). In the USA, a significant proportion of the population utilises X as a primary source of daily news. 48% of active users claim to use X primarily for news purposes (Duarte 2025). The content of these posts mainly includes information about celebrities taking Ozempic and new reports of adverse drug reactions.

In contrast, content creation on Facebook is more evenly distributed among user groups. Thirty-two percent of the posts were created by healthcare professionals, 26% by users who take Ozempic, 24% by influencers, and 18% by news agencies.

### Gender and age of the creators

**Fig. 4:**
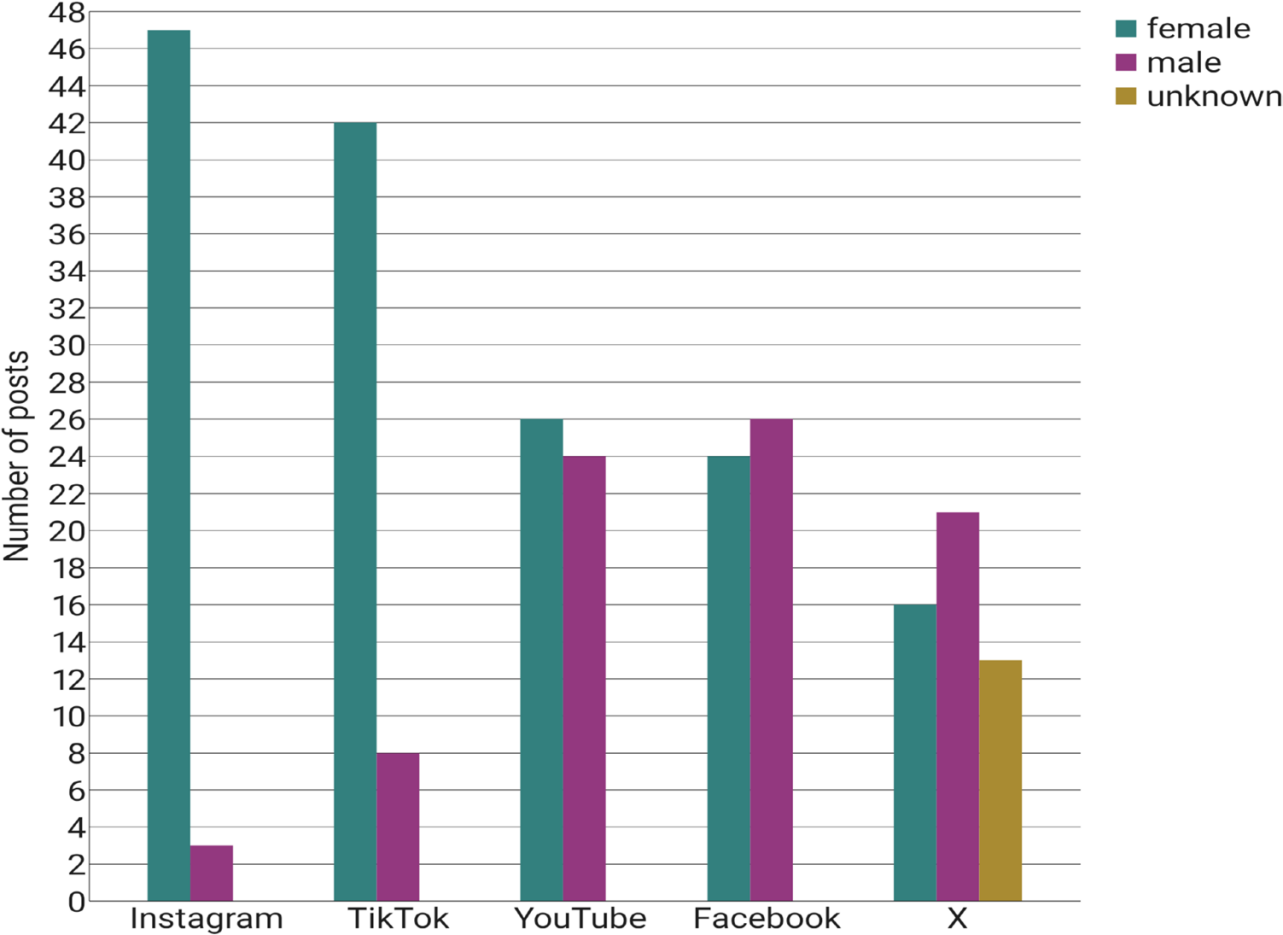
Gender of all creators as a bar chart

Figure 4 shows the gender of all post creators as a bar chart. The posts on YouTube, Facebook, and X were created almost equally by female and male creators; although on Facebook and X, the proportion of male creators slightly predominates. On the platform X, the gender of the creators could not be determined for 26% of the posts and therefore remains unknown. In contrast, 47 of the Instagram posts (94%) and 42 of the TikTok posts (84%) were created by women. It is estimated that the majority of female content creators are between 20 and 45 years of age.

### Application areas

**Fig. 5:**
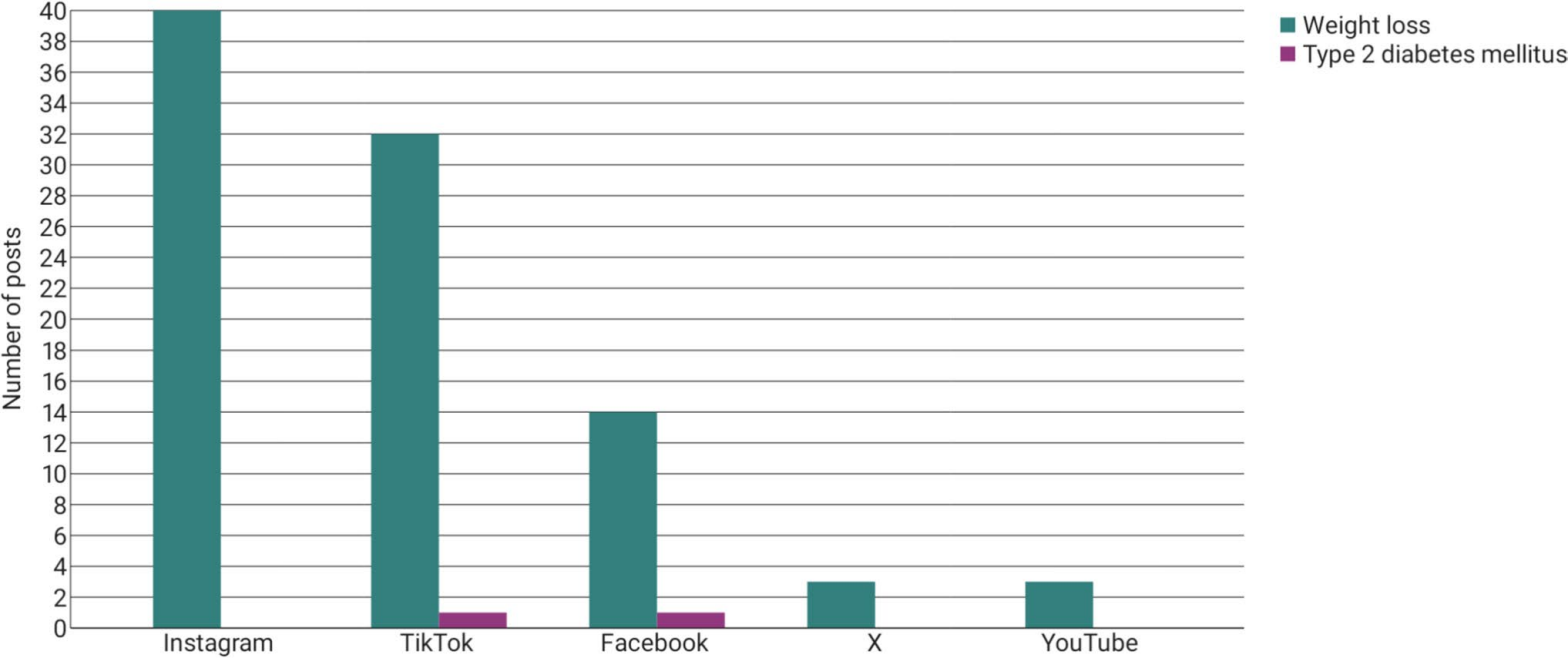
Objective of the use of Ozempic as a bar chart

Ozempic is only approved for glycaemic control in patients with type 2 diabetes mellitus (Rote Liste 2024a, b, c, d). Since 2021 (US) and 2022 (Germany), Wegovy has been approved for weight loss in patients with obesity. Many people seek to utilise semaglutide for weight reduction opt for Ozempic, as it is approximately half the cost of Wegovy (Borsch J, 2024). It is important to note that the application of Ozempic for weight management purposes is considered off-label. Nevertheless, Fig. 5 shows that only 2% of the post creators mentioned taking Ozempic because of their type 2 diabetes mellitus. The remaining 98% stated taking the medication for weight loss or to treat their polycystic ovary syndrome (PCOS).

### Mentioning of adverse events, indications, drug mechanism, supply shortages, and costs

**Fig. 6:**
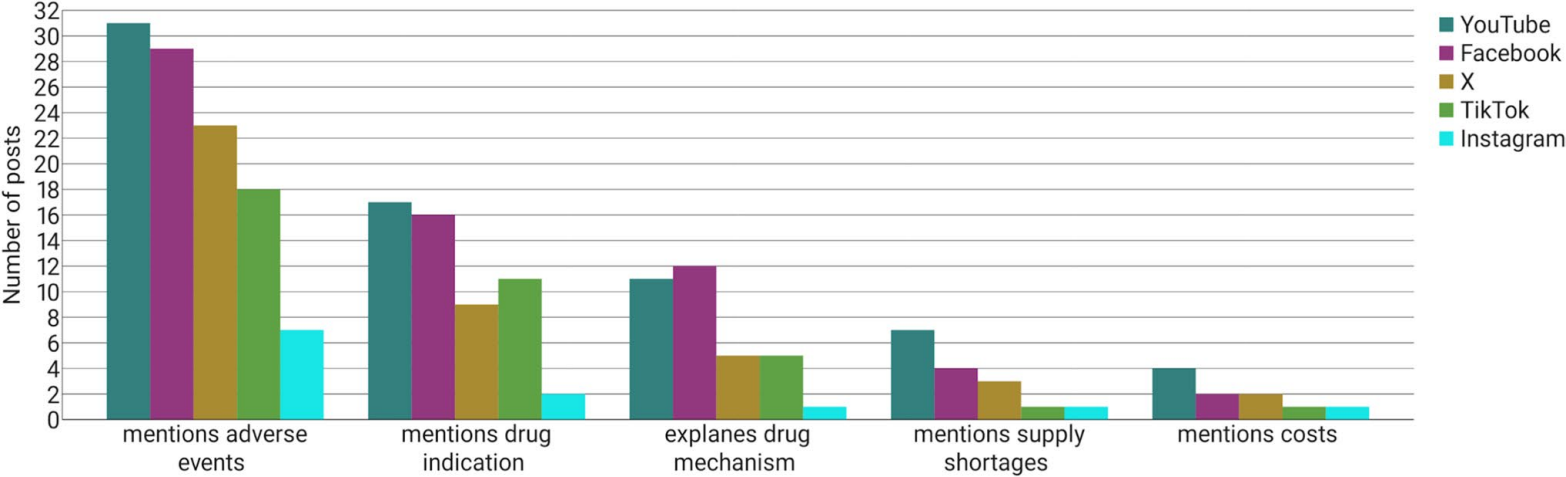
Mentioning of adverse drug reactions, drug indication, drug mechanism, supply shortages, and costs as a bar chart

Figure 6 shows how much information was given in the posts. A total of 43% of the creators mentioned adverse drug reactions that can occur during the semaglutide treatment. Drug indications were mentioned in 22% and the drug mechanism in only 14% of the posts. It is important to understand the mechanism of action of semaglutide in order to better assess its reactions in the body.

In recent years, global supply shortages of Ozempic have been reported (Deutsches Ärzteblatt 2024). Notably, only a total of 6% of the content creators addressed these shortages, despite their importance in ensuring drug supply for patients with type 2 diabetes mellitus. Given that Ozempic has not been approved for weight loss, people have to face high costs taking the medication off-label (Borsch J, 2024). However, information about the treatment costs was only given in 4% of the posts.

Due to the high percentage of healthcare professionals, YouTube provided most information. Adverse drug reactions were mentioned in 62% of the posts found on YouTube. Thirty-four percent mentioned drug indications, and 22% explained the drug mechanism. It is notable that supply shortages were only mentioned in 14% of the posts, while costs were mentioned in a mere 8%.

Very little information was provided by the posts on Instagram because most of the creators only showed pictures of themselves without any text or speech. The presence of a greater number of posts from non-professional users was associated with a lower information content. Only 14% of the Instagram posts contained information regarding adverse drug reactions and an even smaller percentage gave information about drug indications (4%), mechanism (2%), supply shortages (2%), or costs (2%).

To further quantify these findings, a scoring system was employed to evaluate each post. One point was assigned for the mention of each of the following pharmacologically relevant aspects: (1) adverse drug reactions, (2) drug indication, (3) mechanism of action, (4) supply shortages, and (5) treatment costs. This resulted in an overall information score ranging from 0 to 5.

A violin plot was constructed to visualise the distribution of information scores across all platforms. Posts on Instagram demonstrated the lowest overall information content, with a mean score of 0.22 (SD = 0.58) and a median of 0, suggesting a predominance of content lacking pharmacological detail. TikTok posts exhibited slightly elevated values (mean = 0.72, SD = 0.88; median = 0.5), yet continued to indicate a limited presence of medically relevant information.

**Fig. 7:**
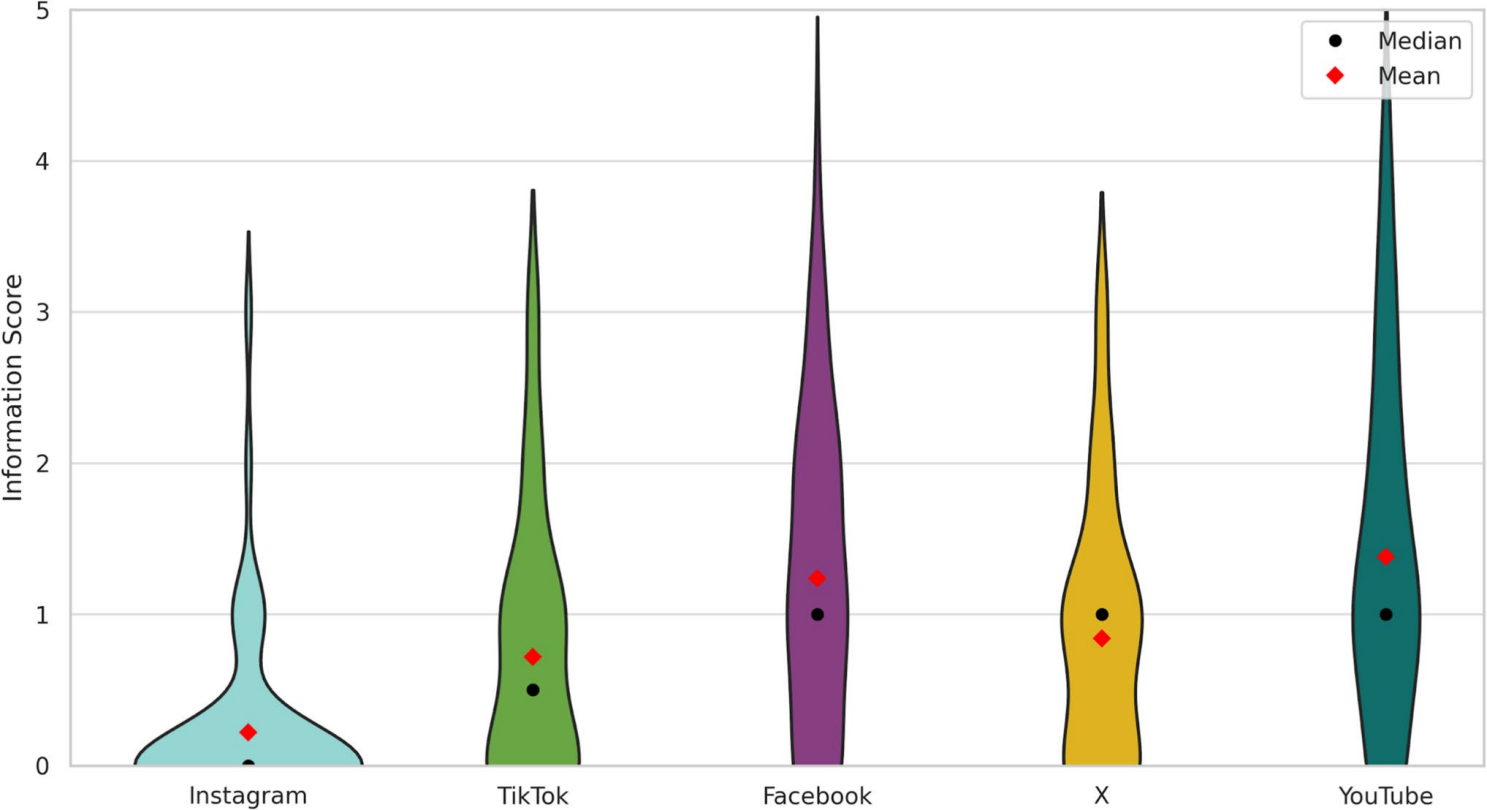
Distribution of information content (0–5 scale) across all analysed posts per platform as a violin plot

In contrast, the mean information score for posts on X was 0.84 (SD = 0.87; median = 1), while Facebook posts demonstrated a mean score of 1.24 (SD = 1.04; median = 1), indicative of increased inclusion of clinically pertinent details. YouTube attained the comparatively highest information score, with a mean score of 1.38 (SD = 1.14; median = 1), although remaining at a low level. The information content on all platforms is found to be inadequate, providing a very limited knowledge base regarding the medication (Fig. 7).

### Mentioning of brand names

**Fig. 8:**
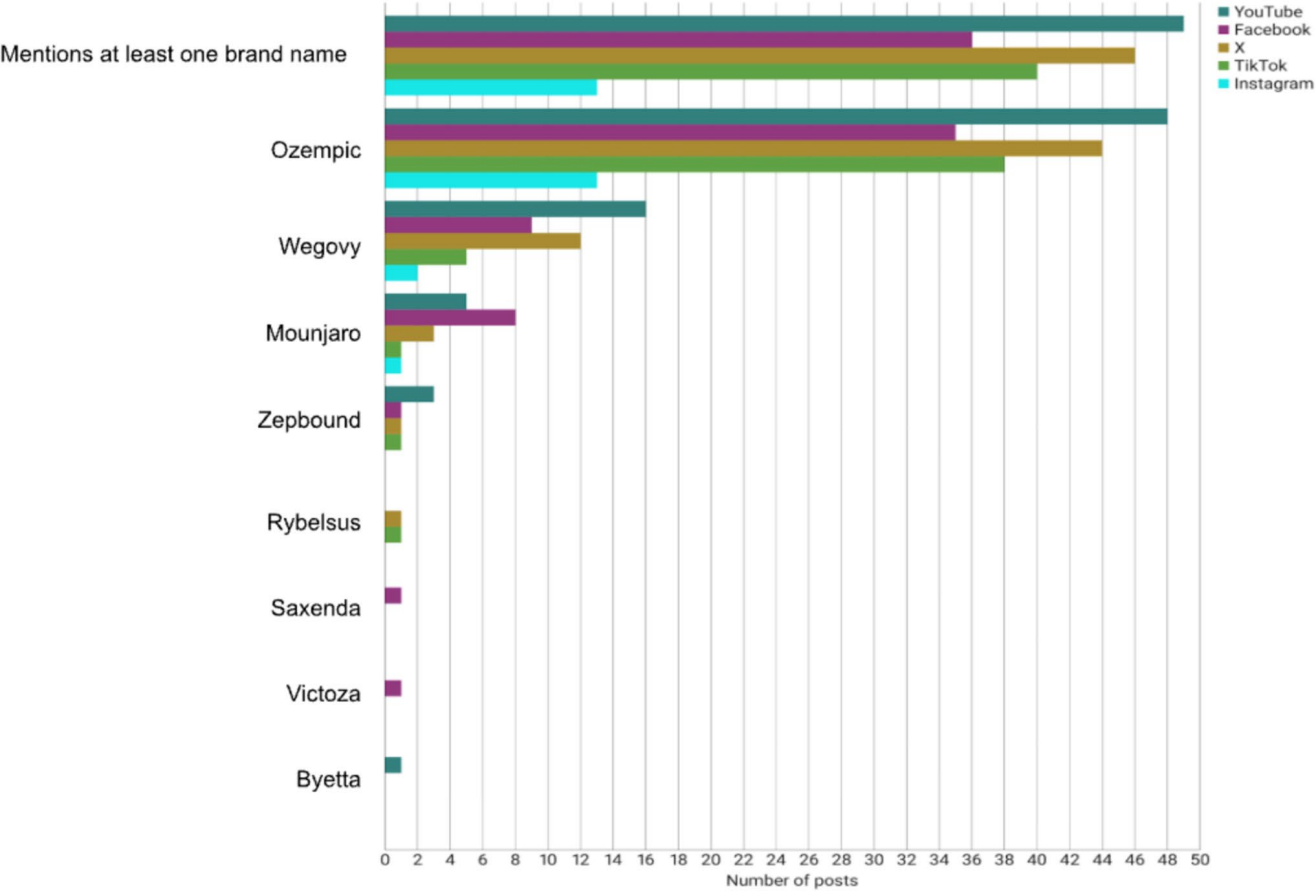
Mentioning of brand names as a horizontal bar chart

Figure 8 shows the mentioning of brand names in the posts. Seventy percent of the creators referenced at least one brand name. Apart from Ozempic, the preparation that is most frequently mentioned is Wegovy, which contains the same active ingredient as Ozempic. Wegovy was mentioned in 44 posts (18%). Rybelsus, an oral preparation that also contains semaglutide, was mentioned in only two posts (1%).

Mounjaro and Zepbound contain tirzepatide, which is another GLP-1 receptor agonist. Mounjaro was mentioned more frequently than Zepbound, particularly on Facebook. Mounjaro was mentioned in 18 posts (7%), while Zepbound was mentioned in 6 (2%). Zepbound was mentioned exclusively in English-language posts. German creators also referenced Mounjaro, but did not discuss Zepbound.

Saxenda and Victoza were each mentioned in only one post. Both medications contain liraglutide. Saxenda has been approved for weight loss in people with obesity, while Victoza has been approved for the treatment of type 2 diabetes mellitus. Byetta is the oldest GLP-1 receptor agonist and has been approved for type 2 diabetes mellitus since 2006. It contains the active ingredient exenatide and was also mentioned in only one post. On Instagram, brand names were only mentioned in 13 posts (26%) because the content creators mostly showed pictures of themselves before and after their weight loss without giving any extra information. This has the potential to negatively affect the ability of subscribers to reach informed decisions regarding their medication.

### Adverse drug reactions

**Fig. 9:**
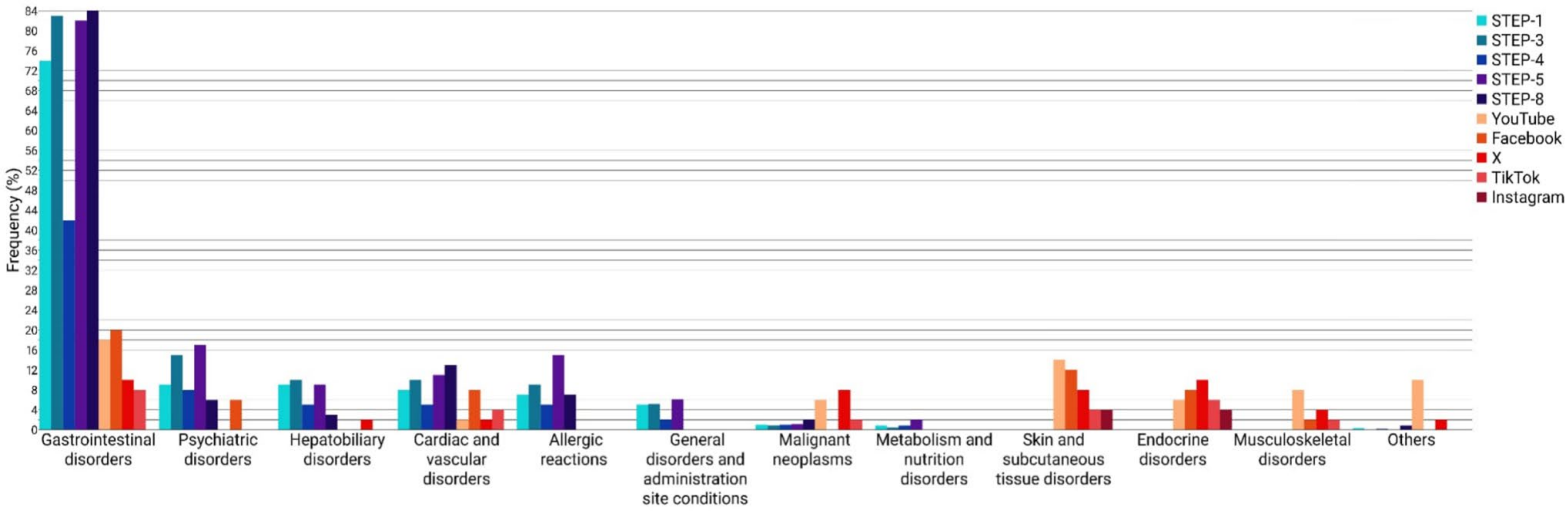
Adverse events mentioned in the posts and in the STEP trials as a bar chart

Adverse drug reactions were mentioned in 43% of the posts. The adverse events (AEs) were classified according to the MedDRA (Medical Dictionary for Regulatory Activities) safety areas of interest. MedDRA, an international medical terminology, facilitates the exchange of information through standardisation. The bar chart in Fig. 9 presents the adverse drug reactions mentioned in the analysed posts in comparison to the findings of the STEP trials. The most frequently reported AEs include gastrointestinal disorders (11%), skin and subcutaneous tissue disorders (8%), and endocrine disorders (7%). Furthermore, cardiac and vascular disorders, malignant neoplasms, and musculoskeletal disorders were mentioned as side effects in 3% of all posts each. Other AEs, such as psychiatric disorders, eye disorders, renal and urinary disorders, immune system disorders, and hepatobiliary disorders, were mentioned only on some social media platforms. Adverse drug reactions were most frequently mentioned on YouTube and Facebook and least frequently on Instagram. Gastrointestinal disorders were not referenced in any Instagram postings.

The results from the social media analysis were compared with the observed adverse drug reactions in the included STEP trials. The STEP (Semaglutide Treatment Effect in People with obesity) programme consists of eight studies with a duration of 68 to 104 weeks that investigated the effect of semaglutide in obese adolescents and adults without diabetes. In the intervention group, participants received 2.4 mg of semaglutide subcutaneously weekly in addition to lifestyle modifications. In the control group, a placebo was administered instead (Kushner RF, 2020). Figure 8 shows the adverse drug reactions observed in the STEP-1, STEP-3, STEP-4, STEP-5, and STEP-8 trials (Wilding et al. 2021; Wadden et al. 2021; Rubino et al. 2021; Garvey et al. 2022; Rubino et al. 2022). Gastrointestinal disorders were observed in an average of 73% of study participants in the intervention group, thus constituting the most prevalent AE. Subsequent to this, the prevalence of psychiatric disorders was recorded at 11%, followed by cardiac and vascular disorders (9%), allergic reactions (9%), hepatobiliary disorders (7%), and general disorders and administration site conditions (4%). Malignant neoplasms and metabolism and nutrition disorders were found in 1% of participants each.

Compared to the STEP-trials, adverse drug reactions are described less frequently on social media. Nevertheless, gastrointestinal disorders are by far the most common AE. Cardiac and vascular disorders, malignant neoplasms, psychiatric disorders, and hepatobiliary disorders are also mentioned in both the STEP-trials and the analysed social media posts.

However, some posts mention AEs that have not been scientifically proven. These include renal and urinary disorders, sore throat, decreased bone density, hair loss, and reduced testosterone production. Furthermore, some of the adverse drug reactions that are listed in the Prescribing Information (PI) were not mentioned in any of the social media posts. These include hypoglycaemia when semaglutide is used concomitantly with insulin, sulfonylureas, or other oral medications for treatment of type 2 diabetes, as well as injection site reactions and anaphylactic reactions (Rote Liste. Ozempic®, 2024b).

### Analysis of particularly popular posts

The extent of the reach of the respective social media posts varies considerably, depending on the specific platform concerned. As the number of views is not typically displayed on Instagram, TikTok, and Facebook, the number of likes is considered the most effective metric for comparing the platforms. Table 2 compares the most liked posts across the five social media platforms included in the analysis. The most popular post on TikTok received the most likes (1,200,000), followed by YouTube (137,807), Facebook (43,650), and Instagram (20,507). The post on X received the fewest likes (9.361). It was observed that the most popular posts were all written in English, as this facilitates a wider reach. Given the heterogeneity of the posts, it is not possible to determine which type of post receives a particularly high level of attention. However, it can be assumed that the most popular posts do not necessarily convey the most information, since the following posts do not include any information pertaining to the drug mechanism, indications, or costs. Instead, content creators reach a large number of followers by not only reporting on semaglutide but also posting videos about fashion or beauty. This phenomenon has the potential to perpetuate unrealistic beauty standards and contribute to the misuse of semaglutide.

**Table 2:** List of the most popular posts found on each of the social media platforms

	**Instagram**	**TikTok**	**Facebook**	**X**	**YouTube**
Views	Unknown	Unknown	Unknown	499,000	3,587,384
Likes	20,507	1,200,000	43,650	9361	137,807
Comments	1108	6421	1	644	1996
Shares	Unknown	6308	16,300	1900	Unknown
Language	English	English	English	English	English
Type of post	Before/after video	Before/after video	Informative video	Experience report	Interview
Probability of AI-modification	93.9%^1^	16.1%^1^	n/a^2^	n/a^2^	n/a^2^
Creator	User who takes Ozempic for weight reduction	User who takes Ozempic for weight reduction	Physician	User who takes Ozempic for weight reduction	Physician
Gender	Female	Female	Male	Female	Male
Mentioning of indications	No	No	No	No	No
Mentioning of AEs	No	No	Yes (interaction with oral medications)	Yes (organ failure)	Yes (reduction of muscle mass)
Mentioning of supply shortages	yes	no	no	no	no
Mentioning of drug mechanism	no	no	no	no	no
Mentioning of costs	no	no	no	no	no

^1^The probability of AI modification was calculated using the software programme “DeepFake-o-meter” (see section *Modification of posts by AI* for an explanation of the functionality of the software)

^2^*n/a* not accessible, because no before-and-after pictures are shown

### Incorrect statements in the posts

In 24 out of 250 posts (10%), incorrect information regarding semaglutide was observed. Incorrect statements were found across all social media platforms. Many of the incorrect statements discuss the application areas of Ozempic and Wegovy. Table 3 shows some examples of incorrect statements found in the analysed posts.

**Table 3:** Examples of incorrect statements found in the analysed posts

Social media platform	Incorrect statement	Correction
Facebook	“Semaglutid ist eins der […] sogenannten GLP-Antagonisten” (“Semaglutide is one of the […] so-called GLP antagonists”)	Semaglutide is a GLP-1 receptor agonist (Papakonstantinou et al. 2024)
Facebook	“die gibt´s noch nicht als Tablette die Wirkstoffe” (“the active ingredients are not available as tablets”)	Since 2020, semaglutide has been available as a tablet under the brand name “Rybelsus” (Rote Liste. Rybelsus®, 2024c)
Facebook	“Ozempic and Wegovy […] are for diabetics”	Wegovy is only approved for weight loss in people with obesity (Singh et al. 2021)
Instagram	“Absolutely safe for health. No reported side effects”	Gastrointestinal disorders are a very common adverse drug reaction (Rote Liste. Ozempic®, 2024b)
Facebook	“with Ozempic comes the aging of the face”	Massive weight loss can lead an older appearance regardless of the cause (Jafar et al. 2024)
YouTube	“Ozempic face is a thing”	This AE can occur by any rapid weight loss in combination with slow elastin turnover (Carboni et al. 2024)
Facebook	“Ozempic, die Fett-weg-Spritze” (“Ozempic, the fat-removal injection”)	Ozempic is exclusively approved for type 2 diabetes (Rote Liste. Ozempic®, 2024b)
X	“die Abnehmspritzen Ozempic, Wegovy und Mounjaro” (“the weight-loss-injections Ozempic, Wegovy and Mounjaro”)	Ozempic is exclusively approved for type 2 diabetes (Rote Liste. Ozempic®, 2024b)
X	“Wegovy und Ozempic, die Abnehmspritzen” (“Wegovy and Ozempic, the weight-loss-injections”)	Ozempic is exclusively approved for type 2 diabetes (Rote Liste. Ozempic®, 2024b)
TikTok	“using the Ozempic dupe to treat my PCOS!”	Ozempic is exclusively approved for type 2 diabetes (Rote Liste. Ozempic®, 2024b)
Facebook	“2/3 of the weight you lose will be muscle”	Semaglutide displays potential for weight loss primarily through fat mass reduction (Bikou et al. 2024)
Facebook	“25% is muscle loss”	Semaglutide eliminates the increase of muscle atrophy markers in skeletal muscle (Xiang et al. 2023)
YouTube	“taking Ozempic life becomes boring”	Semaglutide is found to have positive impact on psychological well-being (Pantanetti et al. 2024)
Facebook	“Essstörung in einer Spritze” (“eating disorder in a needle”)	Binge eating disorders have improved significantly during semaglutide treatment (Richards et al. 2023)
Instagram	“Home made Ozempic recipe”	Herbal ingredients cannot replace semaglutide injections in patients with type 2 DM (Araj-Khodaei et al. 2024)
X	“people are getting depressed over the Ozempic butt”	Presentations like the “Ozempic Butt” can occur as a result of massive weight loss (O´Neill et al. 2024)
X	“her entire family […] now look like WALKING CORPSES…all bc of ozempic”	Massive weight loss, regardless of the cause, can cause people to look up to 5 years older (O´Neill et al. 2024)
X	“Ozempic is dangerous […] it’s absurd to promote it to prevent heart attacks and cancer?!”	For many people with type 2 DM the benefits of Ozempic outweigh the AEs which are mostly mild to moderate (Smits and Van Raalte, 2021)
Instagram	“It also lowers testosterone, thyroid and growth hormones”	Semaglutide led to an increase in total testosterone (Gregorič et al. 2025)
Instagram	“Ozempic can cause bone densitiy loss”	GLP-1 agonists seem to have a positive impact on bone material. Further research is needed (Herrou et al. 2024)
Instagram	“It makes your heart shrank"	Semaglutide led to improvements in exercise function and reduced symptoms in patients with heart failure (Kosiborod et al. 2023)
Facebook	“Migraines […] all because of a shot”	Semaglutide is not associated with a higher risk of neurological outcomes (De Giorgi et al. 2024)
Facebook	“deswegen hab ich Haarausfall” (“that´s why I have hair loss”)	There is possible association between GLP-1 agonists and alopecia (Godfrey et al. 2025)
YouTube	“Ozempic reduces their longevity”	Ozempic led to improvement of cardiovascular risks in patients with type 2 DM. Cardiovascular complications are a leading cause of morbidity and mortality. (Knudsen and Lau, 2019)
Instagram	“GLP-1 agonists like Ozempic should be used as a temporary assistance”	Clinical guidelines support the use of long-term antiobesity medications for sustained weight reduction (Elmaleh-Sachs et al. 2023)

### Modification of posts by AI

Twenty percent of the analysed posts are before-and-after pictures or videos intended to demonstrate weight loss with semaglutide. Seventy-two percent of these posts were uploaded on Instagram and 19% on TikTok. In recent years, artificial intelligence has become increasingly important on social media. To assess whether the before-and-after content had been created or modified using AI tools, the software “DeepFake-o-meter” (https://www.buffalo.edu/digital-scholarship-studio-network/projects/faculty-projects/DeepFake-o-meter.html; last accessed on April 18, 2025) was applied. The software uses AltFreezing to analyse spatial and temporal artifacts in photos and videos. Detected inconsistencies, such as discontinuities, determine the probability that the posts were modified by AI.

**Fig. 10:**
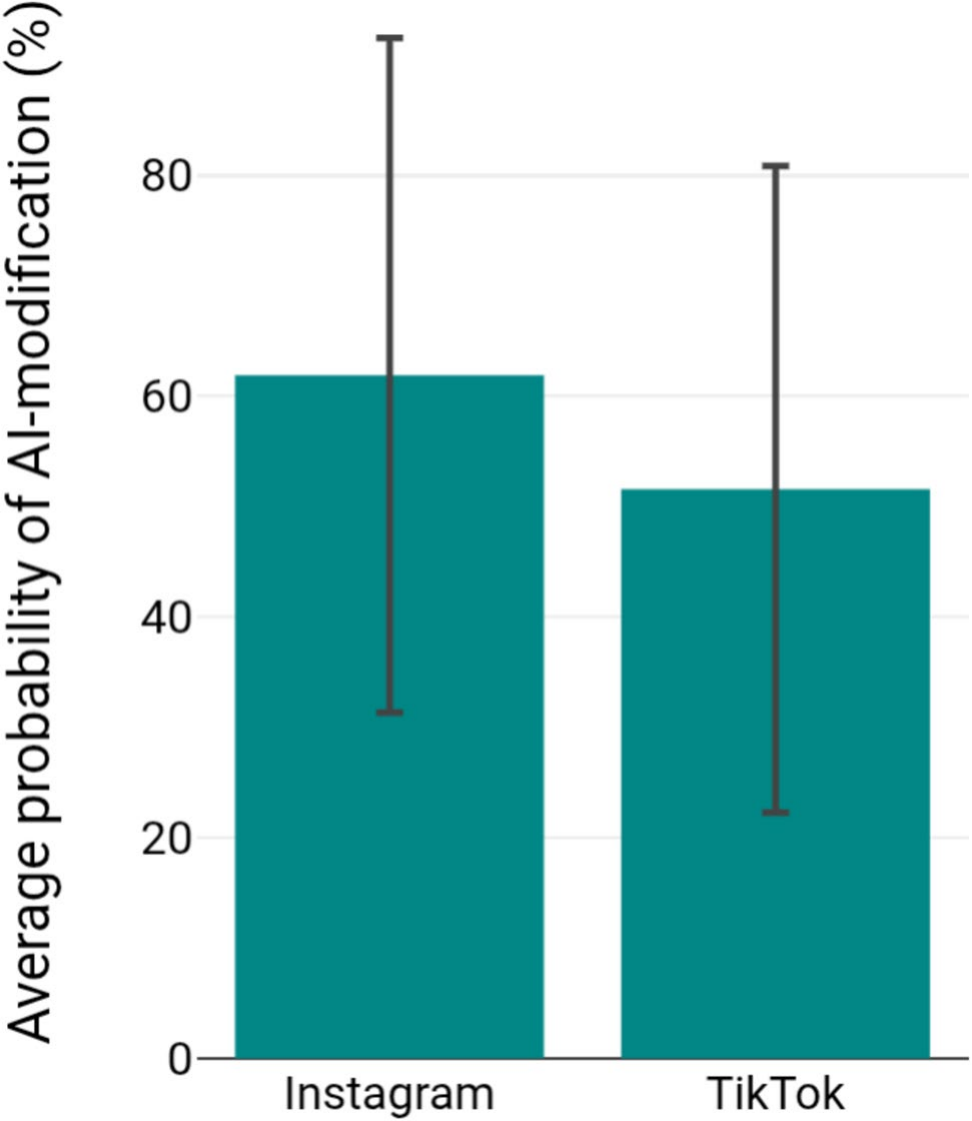
Probability of AI use in before-and-after pictures and videos

Figure 10 presents the average probability of AI-modified content for Instagram and TikTok. On Instagram, before-and-after posts showed an average AI modification probability of 61.9% (± 30.6%). On TikTok, the average probability of AI modification is slightly lower at 51.6% (± 29.3%) in comparison to Instagram. These values indicate a notably high likelihood of AI involvement, particularly on Instagram. The number of before-and-after videos on other social media platforms (X, Facebook, and YouTube) is insufficient to draw reliable conclusions about the use of AI on these platforms.

### Advertising on social media

On Instagram, various accounts posted the same video, claiming to show weight loss success achieved through products that were advertised on a linked website. The posts were all tagged with the hashtag “Ozempic” even though none of the advertised products contained semaglutide. Instead, the products called “sumatra slim belly” and “BioVanish” contained predominantly herbal ingredients such as valerian, hops, amino acids, and berberine. The promotion under the hashtag “Ozempic” is particularly questionable because berberine can lead to interactions with sulfonylureas, which are often used in diabetes therapy (US National Library of Medicine—MedlinePlus Drugs, 2024). The corresponding accounts are fairly well known, with between 3829 and 62,100 followers.

On Facebook, medications are also advertised under the hashtag “Ozempic” These are prescription drugs such as Wegovy and Ozempic, which actually contain semaglutide as the active ingredient. The company claims to be located in Germany and advertises its drugs with the following slogan: “Der Kauf von Medikamenten zur Gewichtsabnahme ist noch einfacher geworden. Sie haben noch kein Rezept? Das ist kein Problem!” (“Buying weight loss drugs has just become easier. Do not have a prescription yet? That is no problem!”). The HWG (Medicinal Products Act) states that in Germany, the promotion of prescription medications is permitted exclusively to medical practitioners, veterinarians, pharmacists, and individuals who are legally authorised to trade in such medications (Bundesministerium für Justiz, HWG §10). The application of the HWG to online advertising is partly unclear and is in need of improvement. To protect social media users from false medical information, the law needs to be stricter and better controlled (Wafai and Seifert, 2025).

## Discussion

### Platform-based disparities in semaglutide reporting

The portrayal of semaglutide varies significantly across the analysed social media platforms, reflecting distinct differences in both the demographics of content creators and the nature of their contributions. As shown in Fig. 3, Instagram and TikTok are dominated by non-professional users (80% and 64%, respectively). The large proportion of content creators on Instagram and TikTok who report on their personal experiences with taking semaglutide is a matter of concern, as these platforms present a unilateral representation of the drug, significantly influenced by individual experiences. X and Facebook featured a more balanced representation. YouTube stood out with 48% of posts created by healthcare professionals, offering comparatively more pharmacological information, including adverse effects (62%), drug indications (34%), and mechanisms of action (22%). This could be attributed to the fact that YouTube is the only platform that enables healthcare professionals to substantiate their authority by obtaining certification (Graham 2022). Consequently, it is an attractive platform for physicians and pharmacists. YouTube was found to provide the most comprehensive information regarding semaglutide, while Instagram and TikTok exhibited significantly limited information across all categories, especially regarding the explanation of drug mechanism (2% and 10%) and the adverse drug reactions (14% and 36%). The limited pharmacological content on TikTok and Instagram is underscored by the low mean information scores of 0.22 on Instagram and 0.72 on TikTok compared to 1.38 on YouTube. The present findings are consistent with the results of the study conducted by Wantuch and Singleton (2025), who examined the presentation of GLP-1 receptor agonists on TikTok. The study revealed that GLP-1 receptor agonists on TikTok are predominantly discussed by non-healthcare professionals and are primarily used for weight loss rather than the treatment of type 2 diabetes. In contrast, the study conducted by Campos-Rivera et al. (2025), who analysed Spanish-language TikTok posts on semaglutide, found that a significantly higher proportion of posts (56%) were created by healthcare professionals. However, the information content was found to be low, with an average DISCERN score of 29.8 out of 75. This discrepancy may reflect cultural differences between language regions. While the Spanish posts in Campos-Rivera’s study were created more frequently by healthcare professionals, the low DISCERN score indicates that even professional authors may fail to provide balanced information on social media. Further research is required to investigate the differences between English- and Spanish-language posts on GLP-1 receptor agonists more profoundly.

Moreover, Instagram and TikTok contain a notably elevated proportion of female content creators. They often document their weight loss journeys using before-and-after images, which were found to be modified by AI in 62% (Instagram) and 52% (TikTok) of cases. This could be due to the fact that young women are more likely to post about their weight loss journeys because they are more sensitive to ideals of thinness and body dysmorphia (Jiotsa, 2021). A study found that 69% of Instagram users and 70% of TikTok users were under the age of 35, categorising them as a vulnerable group for body dysmorphia (Dixon 2024). Considering the demographic distribution on Instagram and TikTok, it can be dangerous for young subscribers to see these posts about weight loss with semaglutide, as it can affect their body image and may cause eating disorders or unreflected drug use. Although each of the included social media platforms has more male than female users, almost exclusively young women post their experiences with semaglutide. Worldwide, a similar number of men and women are affected by obesity (Radtke 2025). It can be assumed that the gender differences in the contributions on semaglutide are caused by the fact that women are more likely to pursue weight loss interventions (Koceva et al. 2024).

### Off-label use for weight reduction and PCOS

As indicated in Fig. 5, Ozempic is predominantly used by content creators for the purposes of weight reduction or the treatment of PCOS. The application of semaglutide in people diagnosed with PCOS who do not meet obesity criteria remains off-label, although studies demonstrate that many young women could benefit from the treatment. Approximately one-third of women with PCOS present with abnormal glucose tolerance and have a higher risk of obesity and type 2 diabetes mellitus (Schröder AK et al., 2004). Semaglutide was found to result in sustained weight loss of an average of 11.5 kg in 6 months in women with PCOS, as well as improved insulin sensitivity and a normalisation of menstrual cycles (Carmina and Longo, 2023).

Content creators have indicated that semaglutide assists in appetite control. They frequently show their weight loss results and encourage their followers to take Ozempic off-label. On TikTok, a creator disclosed that she had initiated treatment with semaglutide to look better “next week at the beach” Especially young women have a high risk of suffering from body dysmorphia due to social media (Losorelli S, 2025). The dissemination of such content has the potential to create false perceptions among the subscribers, leading them to believe that they should also consider taking semaglutide, despite not meeting the criteria for obesity or diabetes. This can pose a significant health risk to individuals who are unaware of the contraindications or adverse drug reactions associated with semaglutide.

In a study conducted by Butaca et al. (2024), various GLP-1 receptor agonists were examined in relation to the term “weight loss” in Google queries. The study identified semaglutide and tirzepatide as the GLP-1 receptor agonists with the highest proportion of off-label use. The off-label utilisation of semaglutide, therefore, signifies a problematic phenomenon that is exemplified not solely by social media but also by Google searches.

### Disparities in drug popularity

The analysis of brand name mentions reveals disparity in the recognition and popularity of different GLP-1 receptor agonists among content creators. These differences appear to be influenced by various factors, including approval status, dosage forms, and treatment indications.

Despite containing the same active ingredient, Rybelsus is less popular than Wegovy. Rybelsus requires greater adherence due to its requirement of daily administration on an empty stomach, a factor that can be more challenging for some individuals (Wollen R, 2024). Although Mounjaro and Zepbound contain the same active ingredient, Mounjaro was mentioned three times more often than Zepbound in the posts. The mentioning of Zepbound in exclusively English-language posts is probably due to Zepbound being only approved in the USA and not in the EU (Leimbach A, 2023). Furthermore, Mounjaro is the only GLP-1 receptor agonist approved for the treatment of both diabetes mellitus and obesity. This feature renders it a particularly attractive option for obese individuals in the prediabetes stage, as they do not have to change their medication as the disease progresses.

An important factor influencing the varying popularity of GLP-1 receptor agonists is the time of approval. Tirzepatide has been approved for the treatment of type 2 diabetes mellitus in the USA and the EU since 2022. Since 2023, it has also been approved for weight management in people with obesity (Siebenand S, 2023). Liraglutide has been approved for weight reduction and type 2 diabetes mellitus since 2015 (Pharmazeutische Zeitung 2015), whereas exenatide has only been approved for type 2 diabetes mellitus since 2006 (Rote Liste 2024a, b, c, d). In 2023, there was a notable increase in the number of newspaper articles and social media posts discussing tirzepatide. Consequently, individuals are more likely to be familiar with the term “tirzepatide” than “liraglutide” or “exenatide”.

### Misleading health information

Misinformation and incomplete reporting of adverse effects are common on social media, potentially leading to misunderstandings and health risks for users seeking reliable information. Regarding ADRs, it was found that the posts mentioned symptoms as adverse effects of semaglutide use despite a lack of scientific evidence. It is plausible that these symptoms occurred in parallel with semaglutide treatment, yet they were not causally related to it. It can be challenging for individuals without a medical background to distinguish between correlation and causation of the symptoms. However, it is important to acknowledge that social media can also provide valuable insights into previously undocumented ADRs. Lardon et al. (2015) demonstrated that, similar to our findings, ADRs reported on social media can deviate significantly from those listed in the PI. They argued that social media data can potentially reveal new ADRs. Nonetheless, the extraction and interpretation of such information remain methodologically complex due to the heterogeneous and unstructured nature of social media content.

Moreover, certain ADRs documented in the PI and associated with the use of medications for the management of type 2 diabetes were not addressed in the analysed posts. This may be attributed to the fact that the majority of content creators use Ozempic off-label for weight reduction rather than for the treatment of type 2 diabetes mellitus. Consequently, they typically do not combine semaglutide with other antidiabetic drugs. This discrepancy poses a potential risk for individuals with type 2 diabetes who rely on social media for information about semaglutide, as it may present a misleading or incomplete picture of the drug’s safety profile.

### AI-enhanced misperception

The increasing use of AI to modify before-and-after images on social media raises concerns about authenticity and its potential impact on body image and health decisions. The analysis shows that many of the before-and-after comparisons on Instagram and TikTok were modified by AI with a probability of over 50% on average. This can be viewed critically because users are often unable to tell whether a photo or video has been modified with AI. Moshel et al. (2022) found that faces generated by AI are more often perceived as real than unmodified faces of actual people, highlighting the persuasive nature of artificial imagery. Similarly, previous studies have demonstrated that the use of photo-editing applications has a major influence on decisions to undergo medical procedures such as surgeries for body modification. In fact, 42% of patients in a study by Benamor et al. (2024) claimed to have been influenced in their choice to undergo aesthetic surgery by photo-editing applications. These findings suggest that both AI-generated and edited images may shape perceptions and body-related decisions, underlining the psychological impact of seemingly “authentic” visual content. The use of AI in video creation can lead to distorted self-perception among social media users, increasing the likelihood of taking weight loss drugs such as semaglutide without medical indication. To avoid such serious consequences, content creators should be encouraged to clearly mark the use of AI in each of their posts. The European AI Act, passed in July 2024, represents a first step towards mandatory labelling of AI-modified content. However, the implementation and monitoring of this regulation on social media remain unclear (Zeitz Digital 2024).

### Promises by social media influencers

Some influencers used promises of efficacy to convince their subscribers to take semaglutide. Impressive quotations from influencers on Facebook include “Wundermittel” and “Ozempic macht es wie Magic,” which can be translated as “wonder drug” and “Ozempic is like magic,” respectively. An influencer on X recommends her followers to take Ozempic and “enjoy the new life On YouTube, semaglutide was described as “miracle drugs” and a “Christmas miracle”. However, these posts do not inform consumers about the potential adverse drug reactions that can occur during semaglutide treatment. To make an informed decision, it is essential for consumers to know both the positive and the negative effects of semaglutide. The following quote from an influencer should be viewed critically: “Absolutely safe for health. No reported side effects”. This assertion could encourage people to take semaglutide expecting no adverse drug reactions. As a matter of fact, during semaglutide treatment gastrointestinal disorders occur very often (Rote Liste 2024a, b, c, d). People also have to be sensitised towards dangerous adverse drug reactions like pancreatitis or gastroparesis, which can have a profoundly negative impact on health and well-being.

But Ozempic has not been universally welcomed. Some of the social media influencers do not want to support the hype and try to warn their subscribers. On X, influencers stated “Stop taking Ozempic” and “Ozempic is dangerous […] it’s absurd to promote it to prevent heart attacks and cancer?!” These extreme statements do not allow users to form an unbiased opinion about semaglutide. There should be controls on social media platforms to prevent misleading information about medications. Social media platforms should facilitate a balanced and comprehensive understanding of pharmaceuticals, encompassing both the benefits and disadvantages of medications.

#### Social media phenomenon “Ozempic Face”

The term “Ozempic Face” describes the aesthetic facial changes that users have noticed in themselves or others following treatment with semaglutide. The “Ozempic Face” is characterised by reduced skin turgor, numerous facial wrinkles, and accelerated skin aging. In addition to the frequently utilised hashtag “ozempicface” the less prominent terms “ozempicbutt” and “ozempicbody” have emerged to draw attention to similar aesthetic changes after the treatment. More than 10,000 posts have already been posted on Instagram and TikTok under the hashtag “ozempicface”. The posts often refer to popular actors whose faces have undergone significant changes following treatment with semaglutide (Schubert 2024). Plastic surgeons and dermatologists have become aware of the social media phenomenon and are already specifically promoting the treatment of “Ozempic Face”.

The aesthetic alterations of the skin are due to the rapid weight loss caused by semaglutide, which also reduces the subcutaneous fat tissue in the face. Any rapid weight loss, regardless of the cause, can lead to facial changes. Studies have shown that massive weight reduction can cause the body to appear up to 5 years older (O´Neill et al. 2024).

#### Social media phenomenon “Ozempic Babies”

Since 2023, there has been increasing coverage of “Ozempic Babies” in the press and on social media. Women have reported successful conception after years of infertility, attributing this outcome to semaglutide. The Facebook group, titled “I got pregnant on ozempic”, has grown to a membership of over 1300 as of August 30, 2024. Due to the current relevance of the topic, the Drug Commission of the German Medical Association has published an article on the subject. Obesity is associated with an increased risk of anovulatory infertility and miscarriage. As a result of weight reduction through semaglutide, the metabolic situation is improved, which can increase fertility. In addition, vomiting and diarrhoea have been identified as the most common adverse drug reactions associated with semaglutide. Reduced absorption of oral contraceptives may be a consequence of the impaired enterohepatic circulation. Since the safety of semaglutide for the unborn child has not yet been sufficiently proven, the drug is not approved for use during pregnancy. Due to the fact that semaglutide has a long half-life of seven days, it should be discontinued if there is a plan to conceive. To date, no increased risk of malformations or miscarriages has been identified, but reproductive toxicity has been demonstrated in animal studies (Dicheva-Radev et al. 2024). Posts about “Ozempic Babies” could encourage women to use semaglutide to improve their fertility. It is therefore necessary to inform the users about the potential risks to the unborn child via social media.

### Limitations

The analysed posts represent a sample of posts about semaglutide. Creators can add new posts about the topic every day, so it is impossible to include all posts about semaglutide that are currently available in one study. Creators are able to modify or delete their posts at any time. They can also render their posts inaccessible to the public. Moreover, fake accounts or purchased likes can distort the data. On social media, it is not possible to determine which information is real and which is modified. Additionally, the visibility of posts varies according to user behaviour. Posts are not presented in the same order to every user.

Only information mentioned in the posts was included in the analysis. It should be noted that some of the posts were connected to websites or additional videos. The information provided in these supplementary videos or websites could not be included in the analysis. Social media is very fast-moving. Influencers react to trends and constantly adapt their content to attract more followers. The analysis is therefore only a snapshot, and it is impossible to give a complete overview about the current data situation on social media.

## Conclusions

The presentation of semaglutide on social media does not sufficiently correspond with scientific findings, particularly regarding indications and adverse drug reactions. The creators of the posts often have insufficient medical knowledge and yet provide information and suggestions about the drug. However, on the positive side, a systematic analysis of social media posts could reveal previously unknown adverse drug reactions.

Our analysis identified YouTube as the most reliable platform for medical information. Conversely, Instagram and TikTok have been found to be unreliable sources of health-related information. This is particularly worrying as these two platforms are used mainly by young adults and adolescents. It is important to expand health education through social media to reach young people and help them make informed decisions. Social media represents a largely unregulated area regarding the Heilmittelwerbegesetz (HWG). Better control of the HWG should be sought to protect consumers. Furthermore, the analysis shows the importance of social media for supply shortages. Social media has a major influence on the demand for certain medications. It is therefore necessary to be aware of current social media trends in order to adjust the expected demand for medicines. The study also showed that the majority of posts had been modified by AI. As a result, users no longer know which photos and videos are real and which have been modified by AI. Young people, in particular, can be strongly influenced in their body perception. Body dysmorphia due to beauty ideals on social media is already a problem for many young people (Khajuria et al. 2025). Combining these beauty ideals with prescription drugs can be dangerous and may even lead to illegal actions to obtain the drugs. The research also shows that social media can provide approaches for the repurposing of drugs. People share their personal experiences with the medication. This can reveal new areas of application, such as infertility treatment.

### Future studies

Further research needs to be done to analyse the representation of other GLP-1 receptor agonists such as tirzepatide and liraglutide on social media. Certifications for medical professionals on YouTube have significantly improved the quality of the content in terms of the information provided. Therefore, future studies should investigate whether introducing certifications for medical professionals on other social media platforms would also be beneficial in improving the quality of posts. Moreover, future studies should aim to investigate the use of artificial intelligence in posts on different social media channels. It can be assumed that the use of AI will increase in the near future. Therefore, it is important for social media users to be aware of the use of AI in posts to be able to distinguish between real and modified videos. It is also important to monitor the representation of semaglutide on social media over the near future in order to assess demand for the medication and anticipate possible supply shortages. Supply shortages for semaglutide have existed for several years and need to be addressed urgently to ensure that people who depend on the medication are no longer exposed to any risks. Furthermore, the effects of semaglutide on female fertility must be investigated. As first-time mothers age, infertility treatment will become increasingly important in society (Attali and Yogev, 2021). Semaglutide could thus gain a new area of application.

## Supplementary information

## Supplementary information

Below is the link to the electronic supplementary material.

ESM 1 (DOCX 70.2 KB)

## Data Availability

All source data for this study are available upon reasonable request.

## Abbreviations

AE: Adverse event
AI: Artificial intelligence
BMI: Body mass index
EMA: European Medicines Agency
FDA: Food and Drug Administration
GKV: Gesetzliche Krankenversicherung (social health insurance)
HWG: Heilmittelwerbegesetz (Medicinal Products Act)
MedDRA: Medical Dictionary for Regulatory Activities
PI: Prescribing Information
