# Supplementary material for: Misrepresentation of semaglutide in social media
Source: Naunyn Schmiedebergs Arch Pharmacol. 2025 Jul 19;399(1):815–32. doi: 10.1007/s00210-025-04403-5 (PMC12894124; doi:10.1007/s00210-025-04403-5)
Supplement: Supplementary file 1 — (DOCX 70.2 KB) [file 210_2025_4403_MOESM1_ESM.docx]

**Supplemental data**

**Misrepresentation of semaglutide in social media**

**Lara Elisabeth Propfe and Roland Seifert**

**Supplemental Table S1** Basic parameters of all analyzed posts on semaglutide.

| **platform** | **views** | **likes** | **comments** | **shares** | **language** | **gender** | **type of posts** | **mentioning of AEs** | **mentioning of supply shortages** | **mentioning of drug mechanism** | **mentioning of indications** | **mentioning of costs** |
| --- | --- | --- | --- | --- | --- | --- | --- | --- | --- | --- | --- | --- |
| Instagram | n/a | 3011 | 2 | n/a | English | female | before/after video | no | no | no | no | no |
| Instagram | n/a | 2919 | 0 | n/a | English | female | before/after video | no | no | no | no | no |
| Instagram | n/a | 2928 | 0 | n/a | English | male | before/after video | no | no | no | no | no |
| Instagram | n/a | 2961 | 1066 | n/a | English | male | information | no | no | no | no | no |
| Instagram | n/a | 4304 | 3 | n/a | English | female | before/after video | no | no | no | no | no |
| Instagram | n/a | 2907 | 1 | n/a | English | female | before/after video | no | no | no | no | no |
| Instagram | n/a | 2238 | 1017 | n/a | English | female | information | yes | no | no | no | no |
| Instagram | n/a | 3359 | 415 | n/a | English | female | news | no | no | no | no | no |
| Instagram | n/a | 2861 | 0 | n/a | English | female | before/after video | no | no | no | no | no |
| Instagram | n/a | 2811 | 2 | n/a | English | female | before/after video | no | no | no | no | no |
| Instagram | n/a | 2906 | 1 | n/a | English | female | before/after video | no | no | no | no | no |
| Instagram | n/a | 2822 | 0 | n/a | English | female | before/after video | no | no | no | no | no |
| Instagram | n/a | 3903 | 635 | n/a | English | female | news | no | no | no | no | no |
| Instagram | n/a | 2057 | 137 | n/a | English | female | information | yes | no | no | no | no |
| Instagram | n/a | 2790 | 1 | n/a | English | female | before/after video | no | no | no | no | no |
| Instagram | n/a | 2908 | 1 | n/a | English | female | before/after video | no | no | no | no | no |
| Instagram | n/a | 3923 | 2 | n/a | English | female | before/after video | no | no | no | no | no |
| Instagram | n/a | 2688 | 73 | n/a | English | female | information | yes | no | no | no | no |
| Instagram | n/a | 2828 | 0 | n/a | English | female | before/after video | no | no | no | no | no |
| Instagram | n/a | 14779 | 1363 | n/a | English | male | information | yes | no | no | no | no |
| Instagram | n/a | 2839 | 3 | n/a | English | female | before/after video | no | no | no | no | no |
| Instagram | n/a | 2806 | 0 | n/a | English | female | before/after video | no | no | no | no | no |
| Instagram | n/a | 2198 | 116 | n/a | English | female | news | no | no | no | no | yes |
| Instagram | n/a | 2895 | 0 | n/a | English | female | before/after video | no | no | no | no | no |
| Instagram | n/a | 2754 | 0 | n/a | English | female | before/after video | no | no | no | no | no |
| Instagram | n/a | 20507 | 1108 | n/a | English | female | before/after video | no | yes | no | no | no |
| Instagram | n/a | 3827 | 1 | n/a | English | female | before/after video | no | no | no | no | no |
| Instagram | n/a | 2950 | 0 | n/a | English | female | before/after video | no | no | no | no | no |
| Instagram | n/a | 2913 | 1 | n/a | English | female | before/after video | no | no | no | no | no |
| Instagram | n/a | 3899 | 11 | n/a | English | female | before/after video | no | no | no | no | no |
| Instagram | n/a | 2038 | 1 | n/a | English | female | before/after video | no | no | no | no | no |
| Instagram | n/a | 2796 | 0 | n/a | English | female | before/after video | no | no | no | no | no |
| Instagram | n/a | 2325 | 3 | n/a | English | female | before/after video | no | no | no | no | no |
| Instagram | n/a | 2559 | 2 | n/a | English | female | before/after video | no | no | no | no | no |
| Instagram | n/a | 5074 | 90 | n/a | English | female | information | yes | no | no | no | no |
| Instagram | n/a | 2874 | 0 | n/a | English | female | before/after video | no | no | no | no | no |
| Instagram | n/a | 10126 | 85 | n/a | English | female | information | yes | no | yes | yes | no |
| Instagram | n/a | 2017 | 7 | n/a | English | female | before/after video | no | no | no | no | no |
| Instagram | n/a | 7143 | 238 | n/a | English | female | information | yes | no | no | yes | no |
| Instagram | n/a | 3056 | 1 | n/a | English | female | before/after video | no | no | no | no | no |
| Instagram | n/a | 2841 | 1 | n/a | English | female | before/after video | no | no | no | no | no |
| Instagram | n/a | 2843 | 2 | n/a | English | female | before/after video | no | no | no | no | no |
| Instagram | n/a | 2956 | 0 | n/a | English | female | before/after video | no | no | no | no | no |
| Instagram | n/a | 2847 | 0 | n/a | English | female | before/after video | no | no | no | no | no |
| Instagram | n/a | 3045 | 10 | n/a | English | female | before/after video | no | no | no | no | no |
| Instagram | n/a | 2992 | 2 | n/a | English | female | before/after video | no | no | no | no | no |
| Instagram | n/a | 3566 | 0 | n/a | English | female | before/after video | no | no | ion | no | no |
| Instagram | n/a | 2935 | 0 | n/a | English | female | before/after video | no | no | ion | no | no |
| Instagram | n/a | 2802 | 0 | n/a | Englisch | female | before/after video | no | no | no | no | no |
| Instagram | n/a | 2832 | 0 | n/a | English | female | before/after video | no | no | no | no | no |
| TikTok | n/a | 189115 | 2103 | 4230 | English | female | self experiment | no | no | no | yes | no |
| TikTok | n/a | 2159 | 84 | 109 | English | female | experience report | yes | no | no | yes | no |
| TikTok | n/a | 1200000 | 6421 | 6308 | English | female | before/after video | no | no | no | no | no |
| TikTok | n/a | 58216 | 737 | 2673 | English | female | before/after video | no | no | no | no | no |
| TikTok | n/a | 14027 | 353 | 959 | English | female | experience report | no | no | no | no | no |
| TikTok | n/a | 53893 | 260 | 252 | English | female | experience report | no | no | no | no | no |
| TikTok | n/a | 1218 | 668 | 123 | German | female | experience report | yes | no | no | no | no |
| TikTok | n/a | 3777 | 173 | 360 | English | female | experience report | no | no | no | no | no |
| TikTok | n/a | 2346 | 178 | 555 | German | male | report | yes | no | no | yes | no |
| TikTok | n/a | 2248 | 348 | 815 | English | male | information | yes | no | no | no | no |
| TikTok | n/a | 7008 | 366 | 631 | English | female | experience report | yes | no | no | no | no |
| TikTok | n/a | 12351 | 675 | 1460 | English | female | information | yes | no | yes | yes | no |
| TikTok | n/a | 3266 | 346 | 69 | English | female | experience report | no | no | no | no | no |
| TikTok | n/a | 19697 | 514 | 2945 | English | female | interview | yes | no | no | no | no |
| TikTok | n/a | 11826 | 537 | 70 | English | female | experience report | no | no | no | no | no |
| TikTok | n/a | 69586 | 1168 | 2373 | English | female | before/after video | no | no | no | no | no |
| TikTok | n/a | 116493 | 988 | 1074 | English | female | before/after video | no | no | no | no | no |
| TikTok | n/a | 21892 | 258 | 105 | English | female | experience report | no | no | no | no | no |
| TikTok | n/a | 14274 | 233 | 252 | English | female | experience report | no | no | no | no | no |
| TikTok | n/a | 6139 | 125 | 350 | English | female | experience report | no | no | no | yes | no |
| TikTok | n/a | 6487 | 195 | 171 | English | female | experience report | no | no | no | no | no |
| TikTok | n/a | 28344 | 1567 | 3600 | English | female | information | yes | no | no | no | no |
| TikTok | n/a | 4884 | 149 | 151 | English | female | experience report | yes | no | no | no | no |
| TikTok | n/a | 19812 | 1167 | 491 | English | female | experience report | no | no | yes | yes | no |
| TikTok | n/a | 31203 | 184 | 2245 | English | female | interview | no | no | no | no | no |
| TikTok | n/a | 4186 | 131 | 81 | English | female | experience report | no | no | no | no | no |
| TikTok | n/a | 4297 | 294 | 165 | English | female | experience report | yes | no | no | no | no |
| TikTok | n/a | 3663 | 219 | 516 | English | female | before/after video | no | no | no | no | no |
| TikTok | n/a | 99060 | 664 | 2834 | English | female | experience report | no | no | no | no | no |
| TikTok | n/a | 13868 | 267 | 1973 | English | female | interview | no | yes | no | yes | no |
| TikTok | n/a | 2151 | 64 | 1092 | English | female | report | yes | no | no | no | no |
| TikTok | n/a | 16058 | 1031 | 1557 | English | female | experience report | yes | no | no | no | no |
| TikTok | n/a | 9397 | 384 | 1252 | English | female | before/after video | no | no | no | no | no |
| TikTok | n/a | 2174 | 96 | 77 | English | female | before/after video | no | no | no | no | no |
| TikTok | n/a | 17889 | 114 | 337 | English | male | interview | no | no | no | no | no |
| TikTok | n/a | 5095 | 465 | 3029 | German | female | information | yes | no | yes | yes | no |
| TikTok | n/a | 5037 | 375 | 382 | English | female | experience report | no | no | no | no | no |
| TikTok | n/a | 2150 | 127 | 135 | English | female | experience report | no | no | no | no | no |
| TikTok | n/a | 11688 | 331 | 1389 | English | female | information | yes | no | no | no | no |
| TikTok | n/a | 6963 | 385 | 1575 | English | male | experience report | yes | no | no | no | no |
| TikTok | n/a | 3810 | 503 | 462 | English | male | information | no | no | yes | yes | no |
| TikTok | n/a | 2341 | 96 | 142 | English | female | before/after video | no | no | no | no | no |
| TikTok | n/a | 5212 | 254 | 225 | English | female | experience report | yes | no | no | no | no |
| TikTok | n/a | 2011 | 29 | 32 | English | female | experience report | no | no | no | no | no |
| TikTok | n/a | 2528 | 0 | 174 | English | female | information | yes | no | no | yes | yes |
| TikTok | n/a | 3037 | 183 | 858 | English | female | before/after video | no | no | no | no | no |
| TikTok | n/a | 14733 | 509 | 940 | English | female | report | no | no | no | yes | no |
| TikTok | n/a | 5807 | 337 | 289 | English | female | before/after video | yes | no | no | no | no |
| TikTok | n/a | 161974 | 4104 | 15129 | English | male | information | no | no | yes | no | no |
| TikTok | n/a | 9385 | 102 | 3197 | English | male | information | no | no | no | no | no |
| Facebook | n/a | 4079 | 1400 | 467 | German | female | interview | no | yes | no | yes | no |
| Facebook | n/a | 3371 | 711 | 600 | German | female | interview | yes | yes | no | yes | no |
| Facebook | n/a | 7397 | 389 | 686 | English | female | information | yes | no | yes | no | no |
| Facebook | 5600 | 2 | 0 | 1 | German | female | report | yes | no | no | yes | no |
| Facebook | 2800 | 7 | 13 | 0 | German | male | information | no | no | yes | yes | no |
| Facebook | 2300 | 2 | 2 | 0 | German | female | report | no | yes | no | yes | no |
| Facebook | n/a | 43650 | 1 | 16300 | English | male | information | yes | no | no | no | no |
| Facebook | 3300 | 3 | 1 | 0 | German | female | information | yes | no | yes | no | no |
| Facebook | 2200 | 29 | 0 | 1 | German | male | advertisement | yes | no | no | no | no |
| Facebook | 2100 | 8 | 5 | 0 | German | male | self experiment | no | no | no | yes | no |
| Facebook | 5900 | 167 | 186 | 0 | English | female | experience report | no | no | no | no | no |
| Facebook | 2000 | 21 | 5 | 5 | German | male | information | yes | no | yes | yes | yes |
| Facebook | 13700 | 8 | 5 | 0 | German | male | report | yes | no | yes | yes | no |
| Facebook | 2700 | 5 | 0 | 0 | German | female | before/after video | no | no | no | no | no |
| Facebook | 1900 | 1 | 1 | 0 | German | female | report | no | no | no | no | no |
| Facebook | n/a | 1349 | 36 | 50 | German | male | information | yes | no | no | yes | no |
| Facebook | 1400 | 5 | 0 | 1 | German | male | advertisement | no | no | no | no | no |
| Facebook | 1200 | 6 | 0 | 1 | German | male | information | yes | no | no | no | no |
| Facebook | 1000 | 6 | 1 | 0 | German | male | information | yes | no | yes | yes | no |
| Facebook | 1500 | 5 | 0 | 0 | German | female | before/after video | no | no | no | no | no |
| Facebook | 1000 | 1 | 0 | 0 | German | male | information | yes | no | yes | no | no |
| Facebook | 1000 | 2 | 0 | 0 | German | male | news | no | no | no | no | no |
| Facebook | n/a | 2285 | 2800 | 64 | English | female | before/after video | yes | no | no | no | no |
| Facebook | 6100 | 29 | 0 | 4 | German | male | information | no | no | no | no | no |
| Facebook | 1000 | 3 | 0 | 0 | German | male | information | yes | no | no | yes | yes |
| Facebook | n/a | 1783 | 400 | 18 | English | female | experience report | no | no | no | yes | no |
| Facebook | n/a | 1217 | 330 | 21 | English | female | experience report | yes | no | no | no | no |
| Facebook | n/a | 4376 | 324 | 133 | English | male | information | no | no | no | no | no |
| Facebook | n/a | 1781 | 179 | 1 | English | female | news | no | no | no | no | no |
| Facebook | 5400 | 15 | 0 | 3 | German | male | interview | no | no | no | no | no |
| Facebook | n/a | 1081 | 345 | 271 | English | female | report | yes | yes | no | yes | no |
| Facebook | n/a | 3843 | 2100 | 936 | English | male | report | yes | no | yes | no | no |
| Facebook | 5700 | 29 | 1 | 2 | German | male | information | no | no | yes | no | no |
| Facebook | 3100 | 57 | 40 | 0 | English | female | experience report | yes | no | no | no | no |
| Facebook | n/a | 908 | 689 | 44 | English | male | news | yes | no | no | no | no |
| Facebook | n/a | 1337 | 368 | 111 | English | male | advertisement | yes | no | no | no | no |
| Facebook | 1900 | 4 | 0 | 2 | German | female | information | no | no | yes | yes | no |
| Facebook | 1000 | 37 | 0 | 0 | German | male | information | yes | no | no | yes | no |
| Facebook | 2000 | 0 | 4 | 0 | German | female | news | no | no | no | no | no |
| Facebook | 1600 | 15 | 1 | 6 | German | female | information | yes | no | yes | yes | no |
| Facebook | 1730 | 8 | 1 | 0 | English | female | experience report | yes | no | no | no | no |
| Facebook | 2600 | 51 | 27 | 0 | English | female | experience report | no | no | no | no | no |
| Facebook | 1400 | 7 | 0 | 1 | English | female | experience report | no | no | no | no | no |
| Facebook | 1300 | 10 | 0 | 0 | English | female | experience report | no | no | no | no | no |
| Facebook | 3100 | 21 | 2 | 0 | English | male | experience report | yes | no | no | no | no |
| Facebook | 1100 | 22 | 3 | 0 | English | male | experience report | yes | no | no | no | no |
| Facebook | n/a | 1838 | 213 | 303 | English | male | information | yes | no | yes | no | no |
| Facebook | 109000 | 2128 | 0 | 8 | English | male | experience report | yes | no | no | no | no |
| Facebook | n/a | 2329 | 336 | 63 | English | male | information | yes | no | no | no | no |
| Facebook | 324000 | 133 | 28 | 0 | English | female | information | yes | no | no | no | no |
| X | 19700 | 6 | 7 | 3 | English | male | information | no | no | no | no | no |
| X | 3900 | 19 | 0 | 8 | English | male | interview | no | yes | no | yes | no |
| X | 4300 | 63 | 4 | 20 | English | male | information | yes | no | yes | no | no |
| X | 499000 | 9361 | 644 | 1900 | English | female | experience report | yes | no | no | no | no |
| X | 6232 | 64 | 4 | 18 | English | male | experience report | no | no | no | no | no |
| X | 30700 | 300 | 40 | 6 | English | male | before/after video | no | no | no | no | no |
| X | 66900 | 749 | 44 | 257 | English | female | information | yes | no | yes | no | no |
| X | 3000 | 3 | 1 | 2 | English | female | report | yes | no | yes | yes | no |
| X | 10300 | 1 | 3 | 3 | German | female | report | yes | no | no | no | no |
| X | 2100 | 12 | 2 | 3 | English | male | interview | yes | no | no | no | no |
| X | 2000 | 5 | 3 | 1 | English | male | interview | yes | no | no | no | no |
| X | 2500 | 3 | 0 | 2 | English | female | interview | yes | no | Ja | yes | no |
| X | 20600 | 121 | 15 | 9 | German | unknown | news | no | yes | no | no | no |
| X | 20400 | 57 | 1 | 6 | English | male | experience report | no | no | no | no | no |
| X | 64300 | 65 | 40 | 16 | German | unknown | news | no | no | no | no | no |
| X | 8500 | 6 | 3 | 2 | German | male | news | no | no | no | no | no |
| X | 2800 | 36 | 5 | 10 | English | male | information | no | no | no | no | no |
| X | 3300 | 4 | 3 | 3 | English | female | experience report | no | no | no | no | no |
| X | 2400 | 1 | 0 | 1 | English | unknown | news | yes | no | no | no | no |
| X | 3400 | 8 | 1 | 4 | English | female | information | no | no | no | no | no |
| X | 10600 | 38 | 12 | 10 | English | male | advertisement | no | no | no | no | no |
| X | 3200 | 12 | 0 | 4 | English | male | news | no | no | no | no | no |
| X | 4900 | 16 | 0 | 9 | English | unknown | news | no | no | no | no | no |
| X | 2300 | 6 | 1 | 0 | English | female | experience report | yes | no | no | no | yes |
| X | 19200 | 15 | 0 | 23 | English | unknown | news | no | no | no | no | no |
| X | 2400 | 3 | 0 | 1 | English | unknown | news | yes | no | no | no | no |
| X | 1900 | 1 | 0 | 1 | English | unknown | news | yes | no | no | no | no |
| X | 1200 | 1 | 0 | 0 | English | unknown | news | no | no | no | yes | no |
| X | 1400 | 32 | 4 | 10 | English | unknown | news | yes | no | no | no | no |
| X | 1900 | 2 | 0 | 1 | English | male | advertisement | yes | no | no | no | no |
| X | 1800 | 11 | 0 | 2 | English | male | comedy | yes | no | no | no | no |
| X | 1800 | 6 | 3 | 3 | English | male | information | no | no | no | no | no |
| X | 1300 | 7 | 1 | 5 | English | female | interview | no | no | no | yes | no |
| X | 1200 | 16 | 1 | 7 | English | male | news | yes | no | yes | yes | no |
| X | 1900 | 12 | 0 | 5 | English | female | experience report | yes | no | no | no | no |
| X | 1600 | 2 | 1 | 2 | English | female | information | no | no | no | no | no |
| X | 1000 | 3 | 0 | 1 | German | unknown | news | no | no | no | no | no |
| X | 1200 | 3 | 0 | 1 | English | unknown | news | yes | no | no | yes | no |
| X | 1300 | 21 | 1 | 4 | English | male | experience report | no | no | no | no | yes |
| X | 1600 | 14 | 0 | 6 | English | male | news | no | no | no | no | no |
| X | 1500 | 12 | 1 | 5 | English | female | information | yes | no | no | no | no |
| X | 1000 | 8 | 0 | 3 | English | female | interview | no | no | no | no | no |
| X | 1400 | 20 | 2 | 10 | English | male | information | no | no | no | no | no |
| X | 1000 | 0 | 0 | 0 | English | unknown | information | yes | no | no | no | no |
| X | 1800 | 3 | 0 | 0 | English | male | experience report | no | no | no | no | no |
| X | 1600 | 11 | 1 | 19 | English | female | news | no | yes | no | no | no |
| X | 1800 | 3 | 1 | 1 | English | unknown | news | no | no | no | yes | no |
| X | 7700 | 44 | 6 | 15 | English | male | news | yes | no | no | yes | no |
| X | 125000 | 144 | 66 | 35 | English | female | experience report | yes | no | no | no | no |
| X | 3620 | 15 | 3 | 3 | English | female | experience report | yes | no | no | no | no |
| YouTube | 95506 | 1492 | 145 | n/a | English | female | interview | yes | no | no | no | no |
| YouTube | 1617616 | 25934 | 2234 | n/a | English | female | news | yes | yes | no | yes | no |
| YouTube | 199862 | 3575 | 119 | n/a | English | female | news | no | no | no | yes | no |
| YouTube | 95329 | 1193 | 78 | n/a | English | female | information | yes | no | no | yes | no |
| YouTube | 10541 | 179 | 5 | n/a | German | male | information | no | no | yes | yes | no |
| YouTube | 46966 | 1794 | 133 | n/a | German | male | information | yes | no | no | yes | yes |
| YouTube | 2359365 | 106760 | 3921 | n/a | English | male | interview | no | no | yes | no | no |
| YouTube | 570774 | 23807 | 419 | n/a | English | male | information | yes | no | no | yes | no |
| YouTube | 1064188 | 39142 | 2785 | n/a | English | female | information | yes | no | no | no | no |
| YouTube | 474646 | 13692 | 805 | n/a | English | female | information | yes | no | yes | no | no |
| YouTube | 3587384 | 137807 | 1996 | n/a | English | male | interview | yes | no | no | no | no |
| YouTube | 11467 | 458 | 5 | n/a | English | female | interview | no | no | yes | yes | no |
| YouTube | 1000996 | 25025 | 1191 | n/a | English | female | news | no | no | no | no | no |
| YouTube | 605988 | 37276 | 643 | n/a | English | male | information | yes | yes | yes | yes | no |
| YouTube | 1798031 | 53777 | 5200 | n/a | English | female | news | yes | no | no | no | no |
| YouTube | 1582051 | 62240 | 3196 | n/a | English | male | interview | no | no | no | no | no |
| YouTube | 1321256 | 54021 | 1806 | n/a | English | female | information | yes | no | no | no | no |
| YouTube | 335858 | 15279 | 408 | n/a | English | male | information | yes | no | no | no | no |
| YouTube | 24155 | 368 | 26 | n/a | English | female | news | yes | no | yes | yes | no |
| YouTube | 94058 | 6634 | 79 | n/a | English | female | information | no | no | yes | no | no |
| YouTube | 14329 | 355 | 2 | n/a | English | male | information | yes | no | no | no | no |
| YouTube | 302780 | 5095 | 216 | n/a | English | male | information | yes | no | no | no | no |
| YouTube | 381744 | 4897 | 281 | n/a | English | male | information | no | no | no | no | no |
| YouTube | 1490 | 27 | 0 | n/a | German | female | news | no | yes | no | no | no |
| YouTube | 965324 | 66819 | 3174 | n/a | Englisch | male | interview | yes | no | no | yes | yes |
| YouTube | 2790812 | 63245 | 9721 | n/a | Englisch | female | interview | no | no | no | no | no |
| YouTube | 94843 | 1811 | 194 | n/a | English | female | information | yes | no | no | no | no |
| YouTube | 5458 | 95 | 0 | n/a | English | female | information | yes | no | yes | yes | no |
| YouTube | 1378136 | 60632 | 2470 | n/a | English | female | news | no | no | no | no | no |
| YouTube | 2239 | 89 | 11 | n/a | English | malw | information | yes | yes | yes | yes | no |
| YouTube | 5308 | 76 | 7 | n/a | English | male | information | yes | no | yes | yes | yes |
| YouTube | 5812 | 83 | 6 | n/a | English | weiblich | news | yes | no | no | no | no |
| YouTube | 166542 | 6974 | 470 | n/a | German | male | information | yes | yes | no | yes | no |
| YouTube | 3770 | 285 | 10 | n/a | English | male | information | yes | yes | no | no | no |
| YouTube | 6553 | 134 | 11 | n/a | Englisch | female | news | no | yes | no | no | yes |
| YouTube | 173722 | 2703 | 252 | n/a | English | female | news | yes | no | no | yes | no |
| YouTube | 14375 | 204 | 25 | n/a | English | male | information | yes | no | no | yes | no |
| YouTube | 1526 | 44 | 2 | n/a | Englisch | female | information | no | no | no | yes | no |
| YouTube | 5032 | 138 | 9 | n/a | English | female | experience report | no | no | no | no | no |
| YouTube | 342967 | 4067 | 258 | n/a | English | female | before/after video | no | no | no | no | no |
| YouTube | 1670 | 37 | 3 | n/a | English | male | information | yes | no | no | no | no |
| YouTube | 3221 | 47 | 0 | n/a | English | male | information | no | no | no | no | no |
| YouTube | 1368 | 23 | 2 | n/a | English | male | information | yes | no | no | no | no |
| YouTube | 291861 | 4590 | 338 | n/a | English | female | information | yes | no | yes | no | no |
| YouTube | 7275 | 189 | 5 | n/a | English | male | information | no | no | no | no | no |
| YouTube | 5807 | 176 | 8 | n/a | English | female | experience report | no | no | no | no | no |
| YouTube | 4634 | 89 | 2 | n/a | English | female | information | yes | no | no | no | no |
| YouTube | 55880 | 1799 | 82 | n/a | English | male | information | yes | no | no | no | no |
| YouTube | 10991 | 348 | 20 | n/a | English | male | information | yes | no | no | no | no |
| YouTube | 1295 | 9 | 0 | n/a | English | male | advertisement | no | no | no | no | no |

*n/a = not available (information not publicly available)
